# Accuracy of Photoplethysmography-Derived Pulse Rate Variability Compared with Electrocardiography-Derived Heart Rate Variability: A Systematic Review and Meta-Analysis

**DOI:** 10.3390/s26165192

**Published:** 2026-08-17

**Authors:** Shiwen Xu, Hao Liu, Zhengliang Liu, Peng Su, Zhuangzhuang Gu

**Affiliations:** 1 School of Physical Education, Henan Normal University, Xinxiang 453007, China; xushiwen@stu.htu.edu.cn (S.X.); liuzhengliang2024@stu.htu.edu.cn (Z.L.); 2 School of Physical Education, Shangqiu Normal University, Shangqiu 476000, China; liuhao@sqnu.edu.cn; 3 School of Physical Education, Guangdong University of Technology, Guangzhou 510006, China

**Keywords:** photoplethysmography, pulse rate variability, heart rate variability, electrocardiography, wearable devices, RMSSD, SDNN, meta-analysis

## Abstract

Photoplethysmography-derived pulse rate variability (PPG-derived PRV) is increasingly used in wearable and camera-based biomedical sensors as a low-burden alternative to electrocardiography-derived heart rate variability (ECG-derived HRV), but its agreement with ECG-derived measurements remains uncertain. This systematic review and meta-analysis evaluated the accuracy of PPG-derived PRV compared with ECG-derived HRV in healthy or apparently healthy non-clinical populations, focusing on the root mean square of successive differences (RMSSD) and the standard deviation of normal-to-normal intervals (SDNN). Forty-three studies were included in the qualitative synthesis; 33 were summarized narratively, and 10 unique studies provided sufficient data for quantitative synthesis. Eight studies contributed to each RMSSD and SDNN meta-analysis. The pooled absolute standardized error was 0.188 (95% CI: 0.066 to 0.309; I^2^ = 11.69%) for RMSSD and 0.134 (95% CI: 0.014 to 0.255; I^2^ = 0%) for SDNN. Sensitivity analyses supported the robustness of RMSSD findings, whereas SDNN estimates were directionally stable but less robust in statistical significance. Because the quantitative synthesis included only 10 studies and was based predominantly on selected resting or controlled conditions, the pooled estimates should not be generalized to sleep, exercise, stress, or free-living settings or interpreted as evidence of interchangeability.

## 1. Introduction

Heart rate variability (HRV) is widely used as a non-invasive marker of cardiac autonomic regulation and has been applied in sports training, recovery monitoring, sleep assessment, stress evaluation, and general health monitoring [1,2]. Traditionally, HRV is derived from electrocardiography (ECG) through the detection of successive R-R intervals, which remains the reference approach for HRV assessment [1]. However, ECG-based measurement usually requires electrodes, dedicated equipment, and greater technical control, limiting its practicality for repeated field-based, daily, or overnight monitoring. These limitations have increased interest in more convenient approaches for acquiring beat-to-beat variability outside laboratory settings [3].

In sport and exercise settings, HRV is commonly used as an indirect marker of autonomic recovery, training stress, and fatigue accumulation. Reductions in parasympathetically mediated indices, particularly RMSSD, may indicate increased physiological strain or incomplete recovery, whereas stable or improving values are often interpreted together with training load and subjective symptoms as supporting readiness for subsequent training. Accurate and practical HRV monitoring is therefore important for individualized training-load adjustment and recovery management.

In applied sport and exercise settings, several validation studies have examined whether portable, wrist-worn, smartphone-based, or chest-strap systems can support repeated HRV or PRV monitoring before or after exercise and during field-based assessment [4,5,6].

Photoplethysmography (PPG) provides one such approach by estimating beat-to-beat variability from peripheral pulse wave signals. PPG-derived pulse rate variability (PRV) is now available through PPG-based wearable and camera-based sensors, including wrist-worn devices, rings, finger or ear sensors, and selected camera-based systems [3,7]. Although ECG-based wearable systems may offer advantages in measurement accuracy, most consumer-oriented devices rely on PPG because of its greater comfort, wearability, design flexibility, and suitability for continuous daily monitoring. PPG-based devices may also be more acceptable for recreational exercisers and the broader consumer fitness population, for whom ease of use and long-term adherence are essential. This practical distinction reflects a key difference between PPG- and ECG-based wearable monitoring. ECG-derived HRV is obtained from cardiac electrical activity and therefore remains the reference approach, but wearable ECG systems usually require an adequate conductive pathway, such as chest-strap electrodes or intentional contact with watch electrodes. By contrast, PPG uses optical sensing of peripheral blood volume changes and can be embedded into passive wrist-, finger-, or ear-worn devices, making it particularly attractive for repeated daily, overnight, or low-burden monitoring. This practical gap is particularly relevant because training and recovery applications require frequent, low-burden measurements rather than occasional laboratory recordings. Nevertheless, PRV and HRV are not physiologically identical. ECG-derived HRV is based on cardiac electrical R-R intervals, whereas PPG-derived PRV is derived from pulse wave intervals and may be influenced by pulse transit time, vascular tone, peripheral circulation, motion artifacts, sensor contact, skin optical properties, and signal-processing algorithms [7,8]. Therefore, the accuracy of PPG-derived PRV relative to ECG-derived HRV must be evaluated systematically before these measurements are interpreted confidently in training, recovery, and health-monitoring contexts.

Beyond research settings, this validation question has become increasingly important because consumer and commercial PPG-based wearables, including smartwatches, fitness bands, and smart rings, are now widely marketed for HRV monitoring, recovery assessment, training readiness, and lifestyle guidance. In practice, users, coaches, and practitioners may interpret PPG-derived variability indices as if they were interchangeable with ECG-derived HRV. However, the central question is not only whether PPG-derived PRV correlates with ECG-derived HRV, but also how large the measurement discrepancy is, whether the direction of error is systematic, and whether the magnitude of error is acceptable for real-world training and recovery decisions.

As summarized in Table S1, the validation literature now spans several PPG-derived platforms, including wrist-worn wearables, ring-based devices, smartphone- or camera-based approaches, and finger-, ear-, or multi-site PPG systems.

This issue is particularly relevant because HRV-guided training and recovery-monitoring systems usually interpret measurements relative to an individual’s longitudinal baseline rather than as isolated absolute values. For applications such as morning readiness assessment, sleep-related recovery monitoring, or quiet recovery periods between training bouts, the practical value of PPG-derived PRV depends on whether its error is sufficiently small, stable, and directionally predictable to detect meaningful within-person changes over time. Establishing the accuracy and agreement of PPG-derived PRV is therefore a necessary first step before more advanced questions can be addressed, including which recording conditions produce the smallest error, which device types or user characteristics are associated with overestimation or underestimation, and whether context-specific correction algorithms can improve practical usability.

PPG-based wearables are increasingly used for recovery, readiness, and lifestyle monitoring, yet their PRV outputs may be interpreted as interchangeable with ECG-derived HRV. Given the diversity of devices and recording approaches represented in the validation literature (Table S1), evaluation should extend beyond correlation to the magnitude and direction of measurement error. This is particularly relevant when measurements are interpreted relative to an individual’s longitudinal baseline, because systematic or unstable error may distort apparent within-person changes.

Although many studies have examined agreement between PPG-derived PRV and ECG-derived HRV, the evidence remains methodologically fragmented. Studies differ in device type, sensor site, recording condition, reference method, HRV metric, and statistical reporting. Some provide complete mean and SD values for both PPG-derived and ECG-derived measurements, whereas others report only correlation coefficients, Bland–Altman results, ICC, MAPE, or other agreement statistics. A previous meta-analysis evaluated portable HRV devices against ECG, but it included a broad range of device types rather than focusing specifically on PPG-derived PRV [3]. The present systematic review and meta-analysis therefore aimed to quantify the accuracy of PPG-derived PRV compared with ECG-derived HRV, with primary quantitative analyses focused on RMSSD and SDNN under representative resting or baseline conditions.

This heterogeneity is evident across studies using different sensor sites, sampling frequencies, fiducial-point definitions, recording durations, and agreement statistics, which makes direct pooling difficult when complete paired summary statistics are unavailable [9,10,11,12].

## 2. Materials and Methods

### 2.1. Study Design and Reporting

This systematic review and meta-analysis was reported in accordance with the Preferred Reporting Items for Systematic Reviews and Meta-Analyses (PRISMA) 2020 statement [13]. The review was designed to synthesize evidence on the accuracy and agreement of photoplethysmography-derived pulse rate variability (PPG-derived PRV), or other pulse-derived beat-to-beat variability measures, compared with electrocardiography-derived heart rate variability (ECG-derived HRV) reference measurements.

### 2.2. Information Sources and Search Strategy

Electronic literature searches were conducted in PubMed, Web of Science, and Scopus for records published from 1 January 2017 to 1 May 2026. The final search was conducted on 1 May 2026. The search strategy combined terms related to heart rate variability, pulse rate variability, photoplethysmography-derived or portable monitoring devices, electrocardiography-derived reference methods, and measurement validity, accuracy, agreement, reliability, or related comparison statistics.

In addition to database searches, other sources were consulted to identify potentially eligible studies. These included manual screening of reference lists from included studies and relevant review articles, as well as additional records identified during full-text retrieval and citation checking. Complete search strategies for PubMed, Web of Science, and Scopus, including field tags, filters, and limits, are provided in Table S5.

### 2.3. Eligibility Criteria and Study Grouping

Studies were eligible for inclusion if they investigated PPG-derived PRV or pulse-derived beat-to-beat variability in comparison with an ECG-derived HRV reference in healthy or apparently healthy non-clinical populations. Eligible populations included healthy volunteers, physically active individuals, athletes, students, healthy adolescents, or other non-clinical participants without a diagnosed cardiovascular, neurological, metabolic, or other specific clinical condition. Studies involving clinical, patient, mixed, or special populations were excluded unless healthy or able-bodied subgroup data were separately extractable and compatible with the review question.

To improve methodological comparability and reduce outcome-level heterogeneity, eligible studies were required to report at least one of the two most commonly used time-domain HRV/PRV indices: the root mean square of successive differences (RMSSD) and/or the standard deviation of normal-to-normal intervals (SDNN). Studies reporting only frequency-domain, nonlinear, pulse-rate-only, or algorithm-performance outcomes without RMSSD or SDNN were excluded from the qualitative synthesis.

Studies were grouped according to their suitability for quantitative synthesis. Studies were included in the quantitative meta-analysis when they reported extractable paired or comparable summary statistics for PPG-derived PRV and ECG-derived HRV, including mean values with standard deviations or sufficient numerical information to derive a standardized estimation error. Studies that met the eligibility criteria but did not provide complete extractable data for meta-analysis were retained in the narrative synthesis.

### 2.4. Study Selection and Data Extraction

After records were exported from PubMed, Web of Science, Scopus, and other sources, a Python-assisted workflow was used to standardize bibliographic information and identify potential duplicate records based on DOI, PMID, title, and close title matches. Potential duplicate records identified by the script were manually checked before removal.

Two reviewers independently screened titles and abstracts according to the prespecified eligibility criteria. Scripts written and executed in Python 3.12.10 (Python Software Foundation, Beaverton, OR, USA) were used to support record management, text-field checking, duplicate detection, and structured screening logs; however, no record was excluded solely on the basis of automated output. Records judged as potentially eligible by either reviewer were retained for full-text assessment.

Full-text reports were independently assessed by two reviewers using the same eligibility criteria. Reasons for exclusion at the full-text stage were recorded in a structured spreadsheet. Disagreements or uncertain cases were resolved through discussion; when consensus could not be reached, a third reviewer was consulted.

Data extraction was performed independently by two reviewers using standardized spreadsheets. Extracted information included study identification, participant characteristics, PPG-derived device or signal source, ECG-derived reference method, recording condition, body position, recording duration, HRV/PRV metric, device mean and SD, reference mean and SD, and reported agreement statistics. Quantitative data used in the meta-analysis were checked against the original full-text reports before analysis. Any discrepancies in extracted values or effect-size eligibility were resolved through discussion and, when necessary, adjudication by a third reviewer.

### 2.5. Data Items and Representative-Condition Selection

Data items were extracted using standardized spreadsheets. For each eligible study, the following information was collected where available: author, publication year, sample size, participant characteristics, health or training status, PPG-derived device or signal source, ECG-derived reference method, sensor site, recording condition, body position, recording duration, breathing condition, HRV/PRV metric, device mean, device standard deviation, reference mean, reference standard deviation, and reported agreement statistics. Agreement statistics included Bland–Altman bias and limits of agreement, intraclass correlation coefficients, mean absolute percentage error, mean absolute error, root mean square error, Pearson correlation coefficients, and other reported validity or reliability indices.

The main quantitative outcomes were RMSSD and SDNN. These outcomes were selected because they were the most consistently reported time-domain indices across studies and because they are commonly used in short-term HRV and PRV monitoring. Reporting of RMSSD and/or SDNN was required for inclusion in the qualitative synthesis. Other HRV/PRV outcomes, including pNN50, LF, HF, LF/HF, SD1, and SD2, were extracted when reported alongside RMSSD or SDNN but were not used as standalone eligibility criteria.

When studies reported multiple eligible results, one representative effect size was selected per study for each HRV/PRV metric in the primary meta-analysis to reduce statistical dependence. Selection followed a prespecified hierarchy: total-sample data were preferred over subgroup data; healthy or apparently healthy non-clinical data were preferred over clinical or special-population data; resting or baseline measurements were preferred over exercise, recovery, stress, posture-change, free-living, or sleep conditions; standard short-term recordings were preferred over ultra-short recordings when both were available; and supine or seated resting measurements were preferred over standing or transition conditions. When multiple PPG devices, sensor sites, or signal sources were reported under otherwise comparable conditions, the most representative device or sensor site for general field-based monitoring was selected, and the decision was recorded in the extraction spreadsheet.

Studies that did not provide complete comparable mean and standard deviation values for both the PPG-derived measurement and the ECG-derived reference were not included in the standardized-error meta-analysis. No numerical conversion was performed for studies that reported only median and interquartile range, graphical data, agreement statistics without paired means and standard deviations, or correlation-based indices. These studies were retained in the qualitative or narrative synthesis when they otherwise met the eligibility criteria.

### 2.6. Methodological and Reporting Completeness Assessment

Methodological and reporting completeness was assessed for studies included in the quantitative meta-analysis using a customized checklist informed by agreement-reporting guidance [14] and HRV-specific reporting recommendations [1,2]. Because the review evaluated paired method-comparison reporting rather than intervention effects or diagnostic classification, formal intervention and diagnostic-accuracy risk-of-bias tools did not map directly to the review question. A customized approach was therefore used to capture PPG/ECG-specific reporting elements, including sensor site, signal processing, artifact handling, and paired summary statistics, within a single assessment. The checklist was used descriptively and was not treated as a formal risk-of-bias instrument. The assessment was designed to evaluate whether each study provided sufficient methodological and statistical information to support interpretation of PPG-derived PRV accuracy against ECG-derived HRV, rather than to exclude studies from the meta-analysis.

The checklist covered the following domains: participant characteristics and sample size; health, training, or clinical status; PPG-derived device or signal source; ECG-derived reference method; sensor site; recording condition; body position; recording duration; breathing control or breathing instructions; HRV/PRV preprocessing and calculation procedures; artifact correction or PPG signal-quality handling; complete mean and standard deviation data for the PPG-derived and ECG-derived measurements; reporting of agreement statistics, such as Bland–Altman bias and limits of agreement, intraclass correlation coefficients, mean absolute percentage error, mean absolute error, root mean square error, or correlation coefficients; and clarity of whether PPG-derived and ECG-derived measurements were obtained simultaneously or under comparable conditions.

Each item was scored as clearly reported (1 point), partially reported (0.5 points), or not reported or unclear (0 points). The percentage score was calculated as the total item score divided by the maximum possible score. Prespecified descriptive categories were high (≥80%), moderate (60% to <80%), and low (<60%) reporting completeness. Two reviewers independently assessed methodological and reporting completeness. Disagreements were resolved through discussion, and a third reviewer was consulted when consensus could not be reached. The assessment results are summarized in Table S2 and were used to inform the interpretation of the quantitative findings and the narrative synthesis.

### 2.7. Effect Measures and Statistical Synthesis

For each comparison, a study-level signed standardized mean discrepancy, termed the signed standardized error, quantified the direction-sensitive difference between PPG-derived PRV and ECG-derived HRV: signed standardized error = [mean(PPG-derived PRV) − mean(ECG-derived HRV)]/ECG-derived reference SD. Using the ECG SD as the standardizer is analogous to reference-SD standardization such as Glass’s delta, although here it was applied descriptively to paired measurements from two methods rather than to an independent-groups treatment effect [15]. Positive values indicated overestimation of ECG-derived HRV by PPG-derived PRV, whereas negative values indicated underestimation. Values closer to zero indicated smaller average discrepancy between the two measurement approaches. The absolute standardized error (ASE) was defined as the absolute value of the signed standardized error and used as the primary pooled outcome to quantify the magnitude of the study-level standardized difference without cancellation between overestimation and underestimation.

This metric was selected because it provides a scale-normalized estimate of study-level measurement discrepancy across studies that reported RMSSD and SDNN under heterogeneous devices, sensor sites, recording durations, and measurement conditions. Taking the absolute value prevents positive and negative mean differences from cancelling in the pooled analysis. Dobbs et al. previously used the same construction—mean difference between the portable device and ECG divided by the ECG SD, followed by an absolute-value transformation—to synthesize portable HRV validation studies [3]. Signed standardized errors were retained in secondary analyses to preserve direction. However, the absolute-value transformation folds negative estimates onto the positive scale; it therefore removes direction and can yield a positive ASE because of sampling variation even when the underlying signed discrepancy is small. ASE was consequently interpreted as a descriptive magnitude measure without a validated acceptability threshold, not as an agreement or interchangeability statistic.

Traditional Bland–Altman mean bias and limits of agreement were not used as the primary pooled endpoint because individual-level paired data, within-participant differences, and limits of agreement were not consistently available across the included studies. Although Bland–Altman analysis is the preferred method for assessing agreement within individual validation studies [16], the lack of consistently reported paired agreement data precluded its use for meta-analytic pooling. Intraclass correlation coefficients quantify reliability or relative consistency, whereas Pearson correlations quantify association; neither provides the individual-level bias and limits of agreement supplied by Bland–Altman analysis [14,16]. These measures address complementary questions but were not reported consistently enough for primary pooling. ASE is instead a descriptive, study-level summary calculated from aggregate means and SDs. It does not characterize the distribution of paired within-participant differences or provide limits of agreement. Its magnitude depends on the ECG reference SD, and no validated ASE cutoff defines acceptable agreement or interchangeability.

Separate meta-analyses were conducted for RMSSD and SDNN. For each outcome, only one representative effect size per study was included in the primary analysis to reduce statistical dependence. Random-effects models with restricted maximum likelihood estimation were used a priori because substantial clinical and methodological heterogeneity was expected across studies, including differences in PPG sensor site, device type, recording duration, participant condition, preprocessing procedure, ECG reference method, and HRV/PRV calculation method.

Because most studies did not report paired-difference SDs or device-reference correlations, sampling variances were estimated assuming a within-participant correlation of r = 0.50 [15,17]. Because study-specific paired correlations were unavailable, r = 0.50 was selected a priori as a pragmatic moderate central assumption and as the midpoint of the prespecified r = 0.25 to 0.75 sensitivity range; it was not treated as an empirically observed study characteristic. Sensitivity analyses using r = 0.25 and 0.75 tested lower and higher correlation assumptions.

Pooled estimates were reported with 95% confidence intervals. Statistical heterogeneity was assessed using Cochran’s Q statistic, tau-squared, and I^2^. Sensitivity analyses included leave-one-out analyses, alternative assumed within-participant correlation analyses, secondary analyses using signed standardized errors to evaluate the direction of bias, and supplementary Pearson correlation analyses when sufficient data were available to examine whether association patterns were consistent with the primary standardized-error findings. Pearson correlations, Bland–Altman statistics, intraclass correlation coefficients, and other agreement metrics reported by narrative-only studies were summarized descriptively rather than pooled when complete standardized-error data were unavailable.

All meta-analyses were performed in R (version 4.5.2; R Foundation for Statistical Computing, Vienna, Austria) using the metafor package (version 5.0.1) [18]. Forest plots were generated to visualize study-specific and pooled estimates.

### 2.8. Narrative Synthesis

Studies that met the eligibility criteria but lacked complete mean and standard deviation values for both PPG-derived and ECG-derived RMSSD or SDNN measurements were summarized narratively. Narrative synthesis was organized according to available agreement statistics, HRV/PRV metrics, device type, measurement condition, and reference method where possible. Particular attention was given to whether evidence supported acceptable agreement between PPG-derived PRV and ECG-derived HRV under standardized resting conditions and whether agreement appeared less consistent under non-resting or more heterogeneous measurement contexts.

### 2.9. Reporting Bias and Certainty Assessment

Formal assessment of small-study effects or publication bias was not performed because fewer than ten studies contributed to each primary meta-analysis. Under these conditions, funnel-plot asymmetry tests and regression-based tests, such as Egger’s test, are statistically underpowered and may provide misleading results. Trim-and-fill adjustment and meta-regression were also not performed because the number of contributing studies was insufficient for stable estimation. Therefore, potential reporting bias was considered narratively by examining the availability of complete quantitative data, selective reporting of HRV/PRV outcomes, and the extent to which studies reported agreement statistics without the summary statistics required for standardized-error calculation.

The certainty of the evidence was not formally graded using GRADE because the review focused on measurement agreement rather than intervention effects, and because the number of studies contributing to each quantitative synthesis was limited. Instead, confidence in the evidence was interpreted narratively according to the number of contributing studies, sample size, methodological and reporting completeness, comparability of PPG-derived and ECG-derived measurements, consistency of findings, measurement condition, and whether the ECG-derived reference method and HRV/PRV preprocessing procedures were clearly reported. These considerations were used to guide the interpretation of the pooled estimates and the narrative synthesis rather than to exclude studies from the review.

### 2.10. Registration, Protocol, and Amendments

This systematic review and meta-analysis was registered in PROSPERO (registration number: CRD420261426165). The registration record is publicly available in the PROSPERO database. A separate full protocol was not published or uploaded. Methodological refinements, including the eligibility criteria for healthy or apparently healthy non-clinical populations reporting RMSSD and/or SDNN, the handling of incomplete summary statistics, and the selection of representative measurement conditions for quantitative synthesis, are described in the Methods section.

## 3. Results

### 3.1. Study Selection

**Figure 1:**
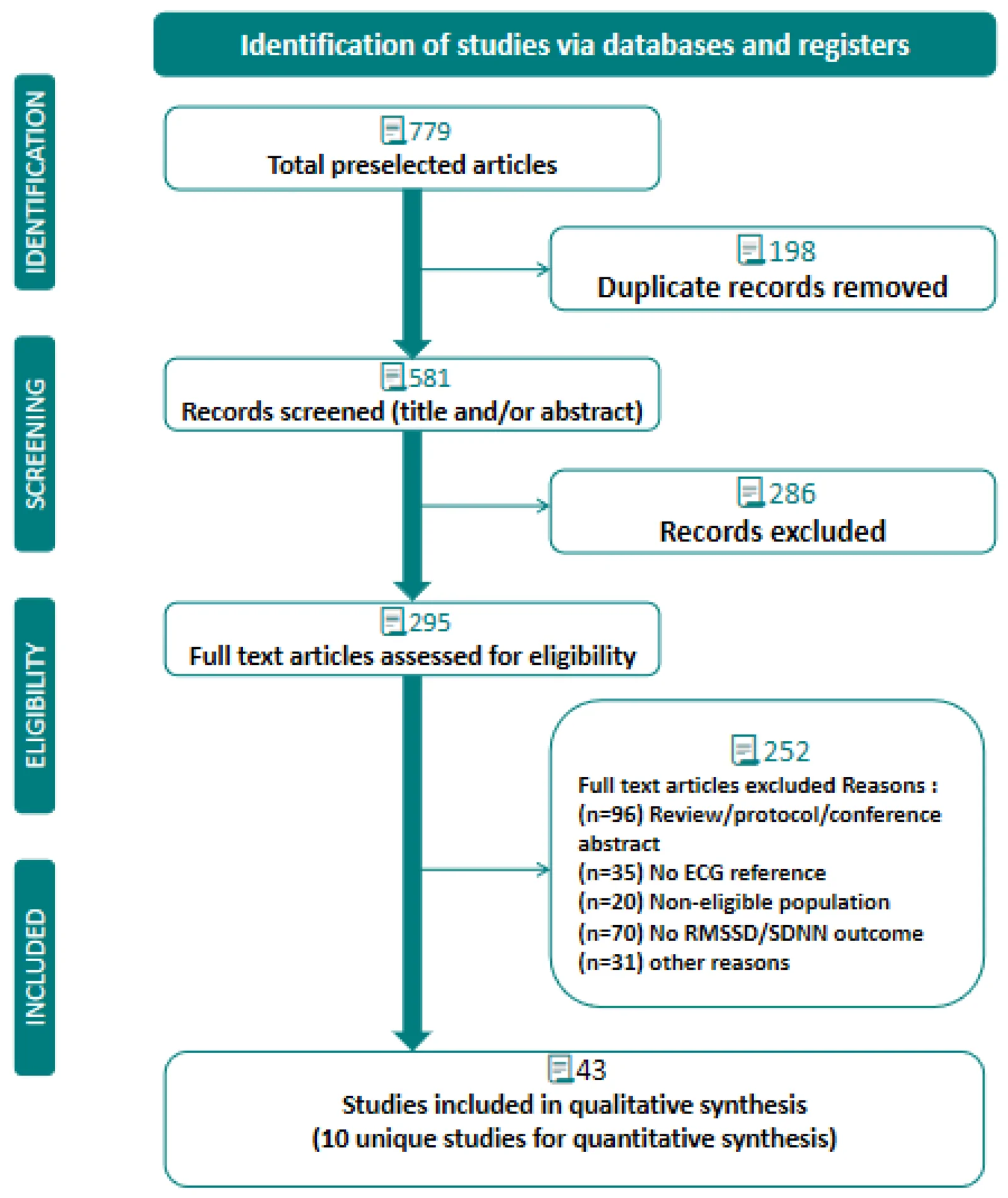
PRISMA flow diagram of study selection.

A total of 779 records were identified from database searches and other sources: PubMed (n = 104), Web of Science (n = 171), Scopus (n = 365), and other sources (n = 139). After duplicate removal, 581 records were screened by title and abstract. Of these, 286 records were excluded, and 295 full-text articles were assessed for eligibility. After full-text assessment, 252 articles were excluded: review, protocol, or conference abstract (n = 96); no ECG reference (n = 35); non-eligible population (n = 20); no RMSSD or SDNN outcome (n = 70); and other prespecified eligibility reasons (n = 31). This left 43 studies in the qualitative synthesis (Figure 1).

Although 43 studies met the eligibility criteria for the qualitative synthesis, only 10 unique studies provided sufficient extractable numerical data for quantitative meta-analysis of RMSSD and/or SDNN. Eight studies contributed to the RMSSD analysis, and eight studies contributed to the SDNN analysis. The remaining 33 studies were retained in the narrative synthesis because they reported RMSSD and/or SDNN but provided incomplete mean and standard deviation data, reported correlation or agreement statistics only, used non-comparable reporting formats, or involved measurement conditions that were not suitable for pooled standardized-error analysis.

### 3.2. Study Characteristics

A total of 43 studies were included in the qualitative synthesis. The included studies primarily involved healthy or apparently healthy non-clinical participants, including adults, athletes, physically active participants, and healthy adolescents, with one mixed-population study retained only because compatible able-bodied subgroup data were separately extractable. All included studies reported RMSSD and/or SDNN. PPG-derived methods remained heterogeneous and included wrist-worn or upper-arm wearable PPG devices, finger- or hand-based PPG sensors, ear or earlobe PPG systems, smartphone or camera-based PPG, ring-based devices, and commercial BVP/PPG wearable systems.

The detailed characteristics of the included studies are provided in Table S1 [4,5,6,9,10,11,12,19,20,21,22,23,24,25,26,27,28,29,30,31,32,33,34,35,36,37,38,39,40,41,42,43,44,45,46,47,48,49,50,51,52,53,54].

Reference methods also varied across studies. Laboratory or clinical ECG systems were the most common reference, but several studies used Holter ECG, chest-strap ECG, wearable ECG sensors, ambulatory ECG, or other ECG-derived R-R/NN interval references. Measurement contexts included controlled resting or baseline assessments, posture-standardized recordings, breathing-guided protocols, sleep or nocturnal monitoring, stress or task-based protocols, exercise or recovery conditions, and selected field-based assessments.

Reported HRV/PRV outcomes focused on the time-domain indices RMSSD and/or SDNN, although some studies also reported additional measures such as pNN50, LF, HF, LF/HF, SD1, or SD2. Agreement and accuracy were reported using diverse statistical approaches, including Bland–Altman bias and limits of agreement, intraclass correlation coefficients, Pearson correlation coefficients, mean absolute percentage error, mean absolute error, root mean square error, and other validity or reliability indices.

Across the qualitative synthesis, studies conducted under resting or otherwise controlled recording conditions generally provided more interpretable comparisons between PPG-derived PRV and ECG-derived HRV. In contrast, wearable, camera-based, smartphone-based, or less standardized recording settings showed greater methodological variability, particularly in sensor site, recording duration, preprocessing procedure, artifact handling, and HRV/PRV calculation method. Among the 43 eligible studies, the 33 narrative-only studies could not be pooled because their results were reported using heterogeneous formats, including correlation-only statistics, Bland–Altman or ICC-only summaries, graphical presentation without complete numerical values, or measurement conditions that were not compatible with the standardized-error framework.

Ten unique studies provided sufficient comparable mean and standard deviation data for at least one RMSSD or SDNN quantitative analysis and were included in the meta-analysis (Table 1). These studies were generally conducted under resting, baseline, or otherwise controlled conditions and contributed to the primary standardized-error analyses. The distinction between qualitative inclusion and quantitative pooling was based on data extractability and comparability rather than study relevance: studies without complete paired or comparable summary statistics were retained for narrative synthesis but not pooled quantitatively.

### 3.3. Narrative Synthesis of Studies Not Included in the Standardized-Error Meta-Analysis

Thirty-three eligible studies were retained for narrative synthesis because they did not provide complete comparable RMSSD or SDNN mean and standard deviation values under representative conditions for standardized-error meta-analysis. The most common reason for narrative-only inclusion was reporting frequency-domain or mixed HRV/PRV metrics alongside RMSSD or SDNN without complete comparable RMSSD/SDNN summary data (n = 11). Other studies reported agreement statistics without complete mean and standard deviation data (n = 7), were conducted under non-resting daily, sleep, nocturnal, or free-living conditions (n = 7), lacked complete comparable RMSSD or SDNN mean and standard deviation values (n = 6), or focused on exercise or recovery conditions (n = 2).

The studies retained for narrative synthesis are summarized in Table S3 according to measurement context, device type, reported RMSSD/SDNN outcomes, and available agreement statistics [4,5,6,9,10,11,12,29,30,31,32,33,34,35,36,37,38,39,40,41,42,43,44,45,46,47,48,49,50,51,52,53,54].

The narrative-only studies covered heterogeneous measurement contexts. Four studies were conducted under resting, posture-standardized, or breathing-related conditions; ten involved sleep or nocturnal monitoring; two involved exercise or recovery; three involved stress or task-based protocols; and several others involved mixed, field-based, or insufficiently standardized recording conditions. This heterogeneity in measurement context was a major reason why these studies were not pooled with the primary resting or baseline standardized-error analyses.

Across the narrative synthesis, PPG-derived PRV tended to show more favorable agreement with ECG-derived HRV under controlled resting, posture-standardized, or breathing-guided conditions. In contrast, agreement appeared more variable during sleep or nocturnal monitoring, exercise and recovery, stress or cognitive-task protocols, field-based recordings, and other less controlled conditions. Because the eligibility criteria excluded purely clinical, patient, or special populations, these narrative findings are most applicable to healthy or apparently healthy non-clinical populations.

The reference methods and statistical reporting approaches also varied substantially. Laboratory ECG was the most common reference method, although Holter ECG, chest-strap ECG, wearable ECG sensors, ambulatory ECG, and other ECG-derived references were also used. Many studies reported correlation coefficients, Bland–Altman bias or limits of agreement, intraclass correlation coefficients, mean absolute error, root mean square error, mean absolute percentage error, or other validity indices rather than complete paired PPG-derived and ECG-derived means and standard deviations. These studies were therefore considered important for contextual interpretation of PPG-derived PRV validity but were not included in the primary standardized-error meta-analysis.

### 3.4. Methodological and Reporting Completeness

Methodological and reporting completeness for the ten studies included in the quantitative synthesis is summarized in Table S2. Five studies were categorized as having high reporting completeness and five as having moderate reporting completeness; no study was categorized as having low or unclear reporting completeness. The mean reporting-completeness score was 78.5%, with scores ranging from 70% to 85%. These categories described completeness of reporting and were not interpreted as formal risk-of-bias judgments.

All ten studies clearly reported participant characteristics, sample size, PPG-derived device or signal source, ECG-derived reference method, and complete mean and standard deviation data required for the RMSSD or SDNN standardized-error meta-analysis. Sensor site was clearly reported in eight studies, whereas one study did not report the sensor site and one study provided unclear or insufficient information. Recording condition, body position, and recording duration were clearly reported in seven studies and partially reported in three studies.

The most common reporting limitations concerned HRV/PRV processing and signal-quality procedures. HRV/PRV preprocessing or calculation procedures were clearly reported in only one study and partially reported in nine studies. Artifact correction, missing-data handling, or PPG signal-quality procedures were not reported in nine studies and were only partially reported in one study. Agreement statistics were fully reported in four studies, partially reported in four studies, and not reported in two studies. These findings suggest that the studies included in the quantitative synthesis provided sufficient summary data for meta-analysis, but incomplete reporting of processing and signal-quality procedures limits the interpretability and comparability of the evidence.

### 3.5. Primary Meta-Analysis of Absolute Standardized Error

**Figure 2:**
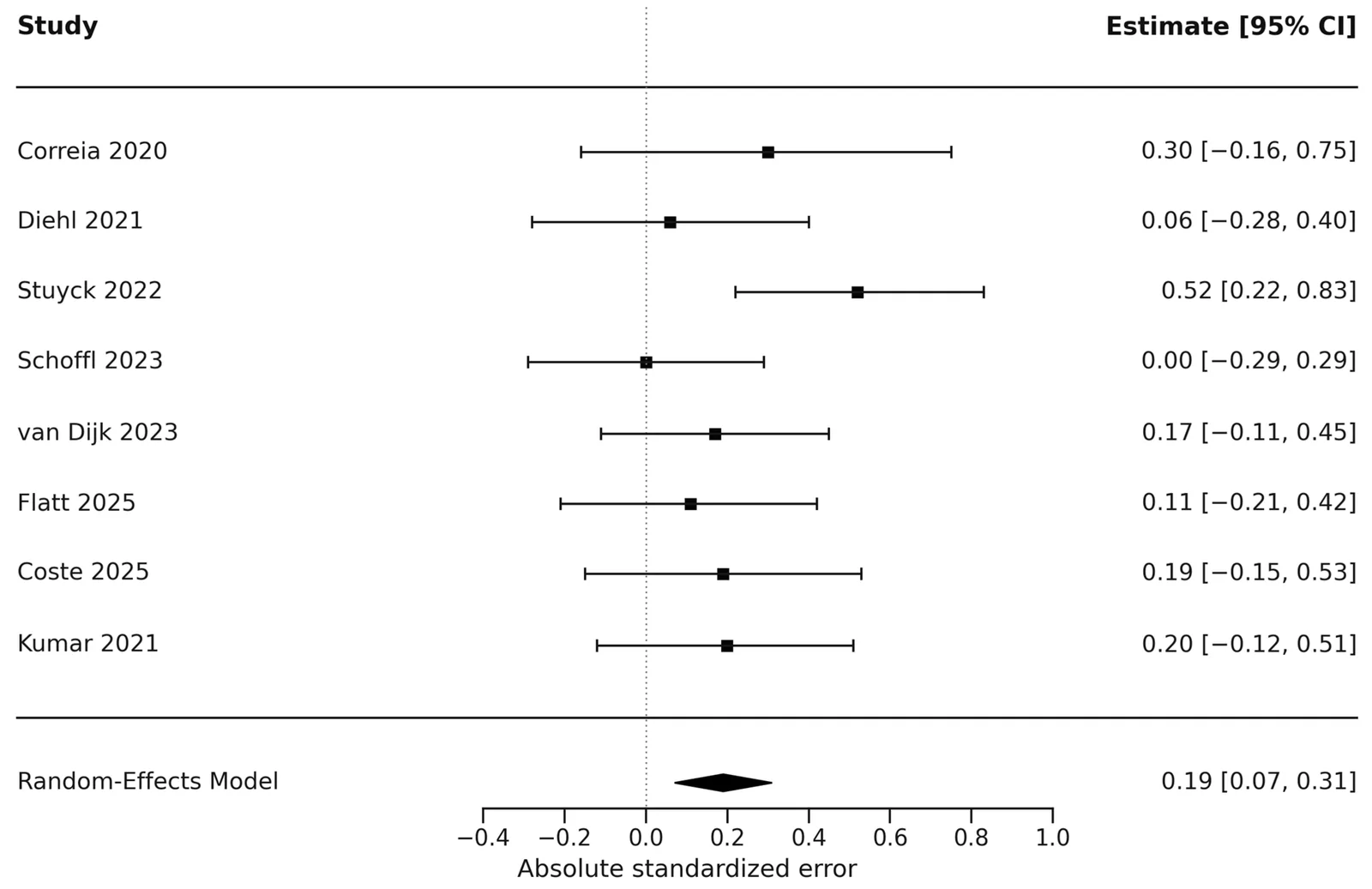
Forest plot of absolute standardized error for RMSSD. Values closer to zero indicate smaller absolute standardized difference between the PPG-derived device and the ECG-derived reference. Squares and horizontal lines show study-specific estimates and 95% CIs; the diamond shows the pooled random-effects estimate [19,20,21,22,23,24,25,28].

Eight studies contributed to the primary RMSSD meta-analysis. The pooled absolute standardized error for RMSSD was 0.188 (95% CI: 0.066 to 0.309) under the representative conditions included in the synthesis (Figure 2) Between-study heterogeneity was low (I^2^ = 11.69%), indicating limited between-study variability in the pooled RMSSD ASE estimate.

**Figure 3:**
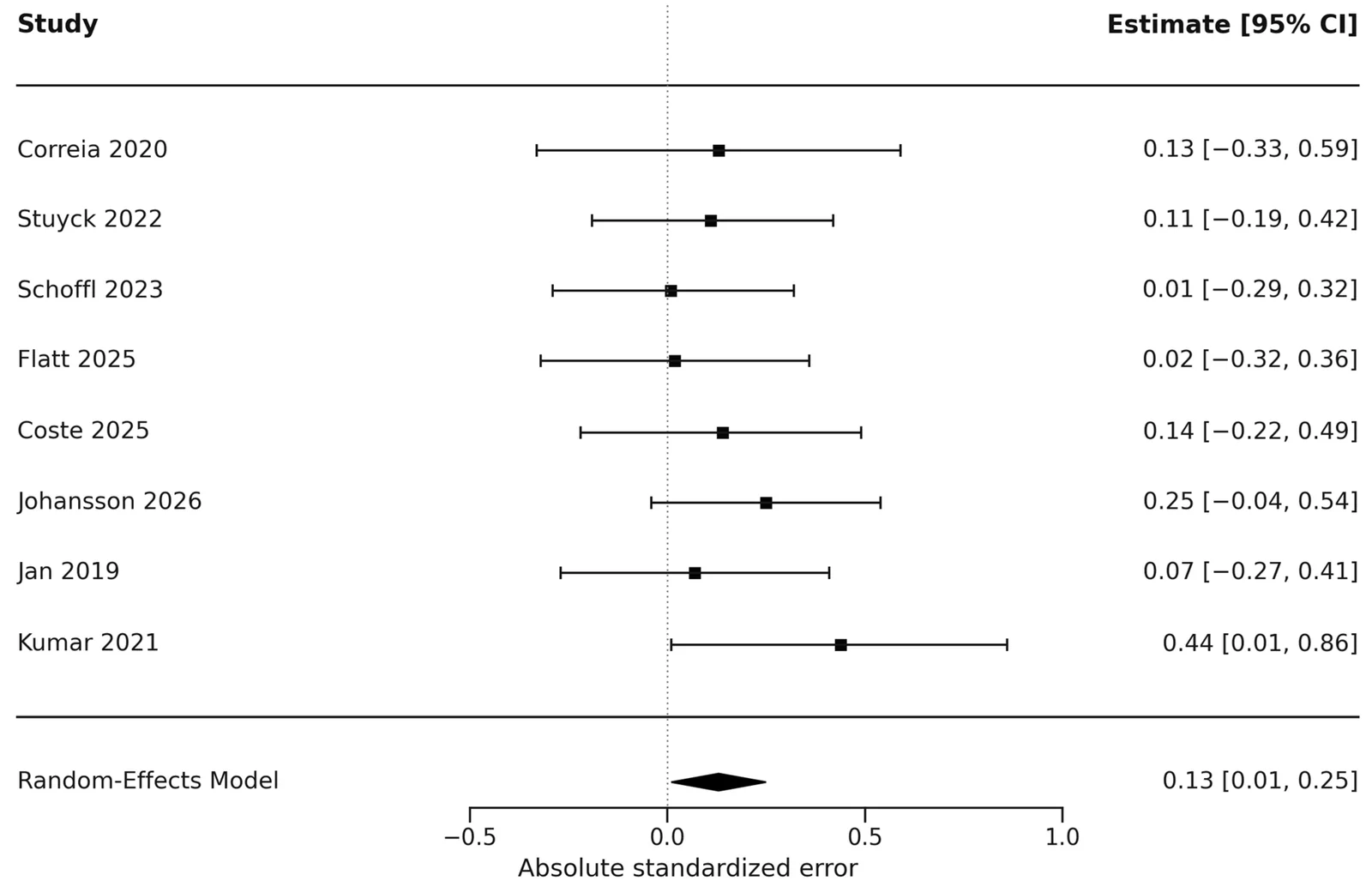
Forest plot of absolute standardized error for SDNN. Values closer to zero indicate smaller absolute standardized difference between the PPG-derived device and the ECG-derived reference. Squares and horizontal lines show study-specific estimates and 95% CIs; the diamond shows the pooled random-effects estimate [19,21,22,24,25,26,27,28].

Eight studies contributed to the primary SDNN meta-analysis. The pooled absolute standardized error for SDNN was 0.134 (95% CI: 0.014 to 0.255) under the representative conditions included in the synthesis (Figure 3) No substantial heterogeneity was observed for SDNN (I^2^ = 0%), indicating limited between-study variability in the pooled SDNN ASE estimate.

Overall, the primary meta-analyses quantified study-level absolute standardized differences between PPG-derived PRV and ECG-derived HRV for both RMSSD and SDNN under selected resting or otherwise controlled representative conditions. However, these findings should be interpreted in relation to the restricted number of quantitatively eligible studies, the use of representative-condition selection, and variability in PPG sources, ECG reference methods, measurement protocols, and signal-processing procedures across studies.

### 3.6. Secondary Signed-Error Analysis

Secondary analyses using signed standardized errors were conducted to examine the direction of bias between PPG-derived PRV and ECG-derived HRV (Table 2). For RMSSD, the pooled signed standardized error was 0.110 (95% CI: −0.054 to 0.275; *p* = 0.188), indicating no statistically significant directional bias. Moderate heterogeneity was observed for the RMSSD signed-error analysis (I^2^ = 51.14%), suggesting that the direction and magnitude of RMSSD bias varied across studies.

For SDNN, the pooled signed standardized error was 0.134 (95% CI: 0.014 to 0.255; *p* = 0.029), indicating a statistically significant tendency for PPG-derived measurements to be higher than ECG-derived reference values in the included studies. No substantial heterogeneity was observed for the SDNN signed-error analysis (I^2^ = 0%).

### 3.7. Sensitivity Analyses

Leave-one-out sensitivity analyses indicated that the RMSSD finding was not driven by any single study. After omitting one study at a time, the pooled absolute standardized error for RMSSD ranged from 0.132 to 0.220, and the overall interpretation remained unchanged. Excluding the breathing-paced condition study also produced a similar pooled estimate (0.192, 95% CI: 0.048 to 0.335), supporting the robustness of the RMSSD result under representative resting or controlled conditions.

For SDNN, leave-one-out analyses yielded pooled estimates ranging from 0.108 to 0.156. The direction and magnitude of the effect remained broadly similar, although statistical significance was attenuated after the removal of some individual studies. This suggests that the SDNN estimate was directionally stable but less robust in statistical significance, likely reflecting the small number of quantitatively eligible studies.

Sensitivity analyses restricted to studies using laboratory or criterion ECG-derived references produced a similar RMSSD estimate (0.178, 95% CI: 0.029 to 0.327). For SDNN, the estimate remained similar in magnitude but was no longer statistically significant when chest-strap ECG-derived references were excluded (0.103, 95% CI: −0.048 to 0.254). These findings support cautious interpretation of SDNN and indicate that reference-method comparability may influence the precision of pooled estimates (Table 3). Sensitivity analyses using alternative assumed within-participant correlations of r = 0.25, 0.50, and 0.75 produced pooled ASE estimates of 0.185 (95% CI: 0.047 to 0.323), 0.188 (95% CI: 0.066 to 0.309), and 0.192 (95% CI: 0.073 to 0.311), respectively, for RMSSD, and 0.137 (95% CI: −0.010 to 0.284), 0.134 (95% CI: 0.014 to 0.255), and 0.128 (95% CI: 0.041 to 0.214), respectively, for SDNN. These estimates were similar across the assumed correlations, indicating that the main conclusions were not materially affected by the variance assumption (Table S6).

### 3.8. Supplementary Exploratory Pearson Correlation Analysis

Pearson correlation coefficients were summarized as supplementary exploratory evidence. In the strict raw-data correlation analysis, six studies contributed RMSSD data, yielding a pooled correlation of r = 0.980 (95% CI: 0.847 to 0.998), with substantial heterogeneity (I^2^ = 97.33%). In the expanded sensitivity analysis after excluding the clinical CABG population study to align with the eligibility criteria, eight studies contributed RMSSD correlation data, yielding a pooled correlation of r = 0.986 (95% CI: 0.924 to 0.997), also with substantial heterogeneity (I^2^ = 97.79%). These high correlations suggest that PPG-derived PRV and ECG-derived HRV generally covaried strongly, but the substantial heterogeneity and the association-based nature of correlation coefficients mean that these findings should not be interpreted as evidence of measurement interchangeability.

## 4. Discussion

### 4.1. General Interpretation and Comparison with Previous Evidence

The primary meta-analyses yielded pooled ASE estimates of 0.188 for RMSSD and 0.134 for SDNN under selected resting or otherwise controlled representative conditions. These values indicate that the absolute differences between the study-level PPG-derived and ECG-derived means were equivalent to 0.188 and 0.134 units of the ECG reference SD, respectively. They quantify average standardized differences at the study level; no validated ASE threshold was applied to classify these estimates as acceptable agreement.

Dobbs et al. previously used the same absolute reference-SD standardized-difference construction in a meta-analysis of portable HRV devices [3]. However, the present review extends this evidence by focusing specifically on PPG-derived PRV rather than portable HRV devices as a broader category. This distinction is important because some portable systems derive interbeat intervals from ECG-based or chest-strap recordings, whereas PPG-derived PRV is based on peripheral pulse-wave detection. Therefore, agreement between PPG-derived PRV and ECG-derived HRV depends not only on device portability, but also on the physiological and technical relationship between cardiac electrical activity and peripheral pulse arrival.

The results also suggest that high correlation between PPG-derived and ECG-derived measures should not be interpreted as direct evidence of measurement interchangeability. Although supplementary Pearson correlation analyses showed strong associations between PPG-derived and ECG-derived RMSSD, correlations describe covariation rather than agreement [14,16]. Similarly, ASE summarizes aggregate study-level mean differences and does not provide individual-participant limits of agreement or reliability estimates such as intraclass correlation coefficients. Taken together with the narrative synthesis, the standardized-error findings therefore support a cautious interpretation: PPG-derived PRV may be useful for estimating HRV-related trends under controlled conditions, but pooled ASE does not establish individual-level agreement or support unrestricted substitution for ECG-derived HRV across all devices, populations, and measurement contexts.

### 4.2. Sensor and Measurement Mechanisms

The observed differences between PPG-derived PRV and ECG-derived HRV are physiologically and technically plausible. ECG-derived HRV is calculated from the timing of cardiac electrical depolarization, usually represented by R-R or NN intervals. In contrast, PPG-derived PRV is calculated from peripheral pulse waves detected optically at the skin surface. Therefore, PPG does not measure cardiac electrical activity directly; it estimates beat-to-beat variability from the arrival of blood volume pulses at a peripheral measurement site.

This distinction is important because the interval between the cardiac electrical event and the peripheral pulse wave is influenced by pulse transit time, vascular tone, arterial stiffness, blood pressure changes, local perfusion, sensor contact, and motion artifact. These factors may introduce variability into PPG-derived interbeat intervals that is not present in ECG-derived R-R intervals. As a result, PPG-derived PRV may approximate ECG-derived HRV under stable resting conditions, but the equivalence between the two signals can weaken when peripheral hemodynamics or signal quality change.

The narrative synthesis supports this interpretation. Agreement appeared more favorable in controlled resting, posture-standardized, or breathing-guided protocols, where movement, posture transitions, and peripheral vascular fluctuations were relatively limited. In contrast, agreement was more variable during sleep or nocturnal monitoring, exercise and recovery, stress or task-based protocols, and free-living conditions. These contexts are more likely to involve changes in autonomic state, vascular tone, movement, sensor pressure, and artifact burden, all of which may affect PPG-derived pulse interval estimation.

These findings suggest that the accuracy of PPG-derived PRV should be interpreted as a property of the complete measurement system rather than the optical sensor alone. Sensor location can alter pulse transit time, local perfusion, and susceptibility to motion or contact artifacts, so agreement observed at one anatomical site may not transfer to another. Lower sampling frequencies reduce temporal resolution and can increase interval quantization or fiducial-point detection error, especially for short-term measures such as RMSSD. Filtering, peak-detection, signal-quality rejection, and missing-beat or artifact-correction algorithms can either reduce noise or introduce device-specific bias into the resulting PRV series [12,31,42]. The limited reporting of artifact handling and signal-quality procedures in the included studies further highlights the need for more transparent sensor-processing descriptions in future validation research.

### 4.3. Practical Implications

From an applied perspective, the findings have direct implications for the use of PPG-derived PRV in consumer wearables, mobile systems, and camera-based monitoring technologies. For these systems, the key issue is not whether PPG-derived PRV perfectly reproduces ECG-derived HRV under all circumstances, but whether its error is small, stable, and directionally predictable enough to support longitudinal within-person monitoring. This distinction is important because HRV-guided recovery and training-readiness systems typically rely on deviations from an individual’s own baseline rather than on a single absolute value.

Under relatively stable conditions, such as resting measurements, morning assessments, or quiet recovery periods, PPG-derived PRV may have greater practical potential if the magnitude and direction of error remain consistent. Sleep-related use should be treated separately and requires context-specific validation because nocturnal movement, changes in sensor contact and peripheral perfusion, and device-specific processing may alter agreement. During movement, exercise, posture transitions, unstable sensor contact, variable breathing, sweating, or rapidly changing vascular states, PPG-derived PRV may be more vulnerable to measurement error because peripheral pulse timing is influenced by both cardiac rhythm and hemodynamic or mechanical factors.

For practitioners and researchers using HRV or PRV in sport, exercise, or health-monitoring settings, these findings suggest that PPG-derived PRV should be used primarily as a standardized, within-device, longitudinal monitoring tool rather than as a universally interchangeable substitute for ECG-derived HRV. Consistent timing, body position, recording duration, breathing instructions, sensor placement, and artifact-control procedures remain essential. PPG-derived PRV should also be interpreted alongside training load, sleep, perceived fatigue, soreness, and other physiological or subjective markers rather than used as a standalone indicator of readiness or recovery.

These considerations are also relevant to the broader practical and translational value of PPG-based wearable systems. The future scalability of HRV-related monitoring in fitness, wellness, and training contexts depends not only on measurement accuracy, but also on user comfort, long-term adherence, and integration into everyday wearable formats. Accurate and reliable HRV-related monitoring is therefore a prerequisite for translating PPG-derived PRV from a consumer-facing wellness feature into a more credible tool for recovery management, training optimization, and health-related monitoring. Future practical and research applications will likely depend on identifying acceptable error thresholds for specific use cases, determining when PPG-derived PRV tends to overestimate or underestimate ECG-derived HRV, and developing device-, sensor-site-, context-, or individual-level correction algorithms.

### 4.4. Limitations

Several limitations should be considered. First, although 43 studies were included in the qualitative synthesis, only a small subset provided complete comparable RMSSD or SDNN mean and standard deviation values for standardized-error meta-analysis. The quantitative findings should therefore be interpreted as estimates based on studies with sufficiently reported and methodologically comparable data, rather than as a complete summary of all available PPG-derived PRV validation evidence. Second, the eligibility criteria intentionally restricted the qualitative synthesis to healthy or apparently healthy non-clinical populations reporting RMSSD and/or SDNN. This improved comparability but limits generalizability to clinical populations, special populations, and HRV/PRV metrics outside RMSSD and SDNN.

Third, methodological heterogeneity and incomplete reporting remain important constraints. The included studies varied in PPG source, ECG reference method, sensor site, recording protocol, and preprocessing approach, while reporting of artifact correction, missing-data handling, and signal-quality procedures was often limited. These factors restrict the ability to determine whether observed differences were attributable to the optical sensor, the physiological difference between peripheral pulse intervals and ECG R-R intervals, or proprietary preprocessing algorithms. Accordingly, the findings are most applicable to resting or otherwise controlled measurement conditions and should not be generalized to sleep, exercise, stress, free-living, clinical, or diagnostic settings without further validation.

### 4.5. Future Research and Reporting Recommendations

Future validation studies should improve the transparency and comparability of PPG-derived PRV assessment. At minimum, studies should clearly report device and sensor characteristics, the ECG-derived reference method, the recording protocol, signal-quality procedures, artifact handling, and HRV/PRV preprocessing algorithms. Whenever possible, PPG and ECG signals should be recorded simultaneously under standardized conditions.

To facilitate future meta-analyses, researchers should report complete paired summary statistics for both PPG-derived and ECG-derived measurements, including means, standard deviations, paired differences, and device-reference correlations where appropriate. Bland–Altman bias and limits of agreement, intraclass correlation coefficients, and error metrics should be reported alongside, rather than instead of, the summary statistics required for quantitative synthesis. Future work should also evaluate PPG-derived PRV across different sensor sites, device types, populations, and real-world conditions to clarify when PPG-derived PRV can be used for longitudinal monitoring and when ECG-derived HRV remains necessary.

Future research should move beyond global device-level validation by estimating device-, condition-, and user-level moderators of bias across resting, sleep, recovery, stress-task, and free-living settings. These data are needed to develop context-aware correction models and define use-specific error thresholds.

## 5. Conclusions

This systematic review and meta-analysis yielded pooled ASE estimates of 0.188 for RMSSD and 0.134 for SDNN under selected resting or otherwise controlled representative conditions. Together with the narrative evidence, these findings support the potential use of PPG-derived PRV only within the devices, populations, and conditions represented in the evidence base. Because the quantitative synthesis included only 10 studies and was based predominantly on selected resting or controlled conditions, the pooled estimates should not be generalized to sleep, exercise, stress, or free-living settings without further validation.

Accordingly, PPG-derived PRV should not be regarded as universally interchangeable with ECG-derived HRV across all devices, populations, and recording contexts. Agreement may be influenced by sensor site, signal quality, artifact correction, pulse transit time, vascular responses, recording condition, and preprocessing algorithms. Future validation studies should improve reporting of signal-processing procedures and provide complete paired summary statistics to support more robust evidence synthesis.

## Figures and Tables

**Table 1:** Studies included in the RMSSD and SDNN quantitative meta-analyses.

Study	Metric	PPG Source	ECG Reference	Condition	n	ASE
Correia (2020) [19]	RMSSD	James One	Polar H10	Rest	18	0.296
Diehl (2021) [20]	RMSSD	PPG sensor	ECG (3-lead system)	Resting Baseline; Sitting; 3 min	94	0.059
Stuyck (2022) [21]	RMSSD	Empatica E4 wristband	Nexus-10 MKII ECG device	Seated resting; Seated; 5 min	39	0.524
Schoffl (2023) [22]	RMSSD	BioSemi™ Active-Two System	BioSemi™ Active-Two System	Baseline (Able-bodied); Seated	41	0.004
van Dijk (2023) [23]	RMSSD	Happitech SDK	NeXus apparatus	Breathing Pace 2: 4–6; Seated; 5 min	57	0.169
Flatt (2025) [24]	RMSSD	Kairos wristband	ECG	Resting; Supine; 3 min	32	0.106
Coste (2025) [25]	RMSSD	Polar OH1	Polar H10	Overall participants; Supine; 5 min	31	0.190
Kumar (2021) [28]	RMSSD	in-house finger photo pulse plethysmography apparatus	analog Lead II ECG amplifier	resting; sitting; 5 min	50	0.199
Correia (2020) [19]	SDNN	James One	Polar H10	Rest	18	0.126
Stuyck (2022) [21]	SDNN	Empatica E4 wristband	Nexus-10 MKII ECG device	Seated resting; Seated; 5 min	39	0.114
Schoffl (2023) [22]	SDNN	BioSemi™ Active-Two System	BioSemi™ Active-Two System	Baseline (Able-bodied); Seated	41	0.014
Flatt (2025) [24]	SDNN	Kairos wristband	ECG	Resting; Supine; 3 min	32	0.020
Coste (2025) [25]	SDNN	Polar OH1	Polar H10	Overall participants; Supine; 5 min	31	0.136
Johansson (2026) [26]	SDNN	CameraHRV app	Polar H10 chest strap	Resting; Supine, reclined at 45°; 60 s	37	0.248
Jan (2019) [27]	SDNN	PPG left hand (LH)	one-lead ECG lead II	Resting I baseline	30	0.068
Kumar(2021) [28]	SDNN	in-house finger photo pulse plethysmography apparatus	analog Lead II ECG amplifier	resting; sitting; 5 min	50	0.435

Note: One representative condition per study and metric was used in the primary meta-analysis. PPG = photoplethysmography; ECG = electrocardiography; RMSSD = root mean square of successive differences; SDNN = standard deviation of normal-to-normal intervals; ASE = absolute standardized error.

**Table 2:** Secondary meta-analysis of signed standardized errors.

Analysis	k	Signed Standardized Error	95% CI	I^2^	*p*	Interpretation
RMSSD	8	0.110	−0.054 to 0.275	51.14%	0.188	Small positive trend; CI crosses 0
SDNN	8	0.134	0.014 to 0.255	0.00%	0.029	Small positive bias; CI > 0

**Table 3:** Meta-analysis and sensitivity analysis results.

Analysis	k	Absolute Standardized Error	95% CI	I^2^	*p*	Interpretation
RMSSD primary	8	0.188	0.066 to 0.309	11.69%	0.0025	Pooled ASE estimate
RMSSD excl. paced breathing	7	0.192	0.048 to 0.335	23.85%	0.0088	Similar
RMSSD criterion ECG only	6	0.178	0.029 to 0.327	22.9%	0.0192	Similar
SDNN primary	8	0.134	0.014 to 0.255	0.00%	0.0293	Pooled ASE estimate
SDNN excl. Johansson	7	0.110	−0.023 to 0.243	0.00%	0.1044	CI crosses 0
SDNN excl. Kiran Kumar	7	0.108	−0.018 to 0.234	0.00%	0.0938	CI crosses 0
SDNN criterion ECG only	5	0.103	−0.048 to 0.254	0.00%	0.1792	CI crosses 0

## Data Availability

The extracted data supporting the quantitative analyses are provided in the Supplementary Materials. Additional analysis files are available from the corresponding author upon reasonable request.

## Supplementary Materials

The following supporting information can be downloaded at: https://www.mdpi.com/article/10.3390/s26165192/s1, Table S1: study characteristics of the 43 included studies; Table S2: methodological and reporting completeness assessment for the 10 studies included in the quantitative meta-analysis; Table S3: narrative synthesis of the 33 studies that did not provide complete comparable mean and standard deviation data for the main RMSSD or SDNN meta-analysis; Table S4: supplementary exploratory Pearson correlation meta-analysis and sensitivity-analysis results; Table S5: complete search strategies for PubMed, Web of Science, and Scopus; Table S6: sensitivity analysis using alternative assumed within-participant correlations; Figure S1: RMSSD signed-error forest plot; Figure S2: SDNN signed-error forest plot; Figure S3: strict exploratory RMSSD Pearson correlation forest plot; Figure S4: expanded RMSSD Pearson correlation sensitivity forest plot. References to all studies reported in the Supplementary Materials are included in the main reference list [4,5,6,9,10,11,12,19,20,21,22,23,24,25,26,27,28,29,30,31,32,33,34,35,36,37,38,39,40,41,42,43,44,45,46,47,48,49,50,51,52,53,54].
