# Supplementary material for: Accuracy of Photoplethysmography-Derived Pulse Rate Variability Compared with Electrocardiography-Derived Heart Rate Variability: A Systematic Review and Meta-Analysis
Source: Sensors (Basel). 2026 Aug 17;26(16):5192. doi: 10.3390/s26165192 (PMC13517957; doi:10.3390/s26165192)
Supplement: Supplementary file 1 [file sensors-26-05192-s001.zip › Supplementary_Figure_S1.pdf]

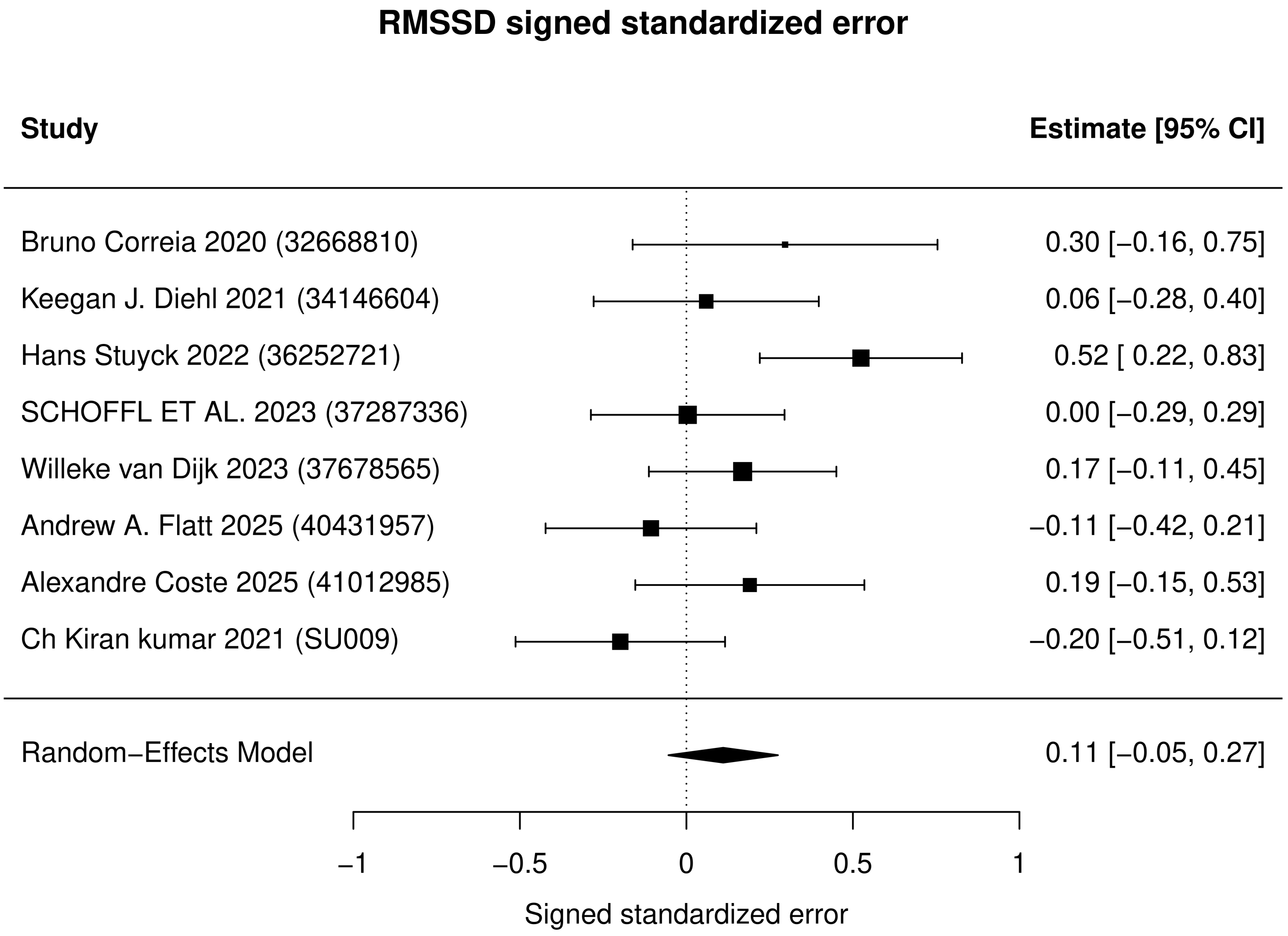
RMSSD signed standardized error
Study
Estimate [95% CI]
Bruno Correia 2020 (32668810)
0.30 [−0.16, 0.75]
Keegan J. Diehl 2021 (34146604)
0.06 [−0.28, 0.40]
Hans Stuyck 2022 (36252721)
0.52 [0.22, 0.83]
SCHOFFL ET AL. 2023 (37287336)
0.00 [−0.29, 0.29]
Willeke van Dijk 2023 (37678565)
0.17 [−0.11, 0.45]
Andrew A. Flatt 2025 (40431957)
−0.11 [−0.42, 0.21]
Alexandre Coste 2025 (41012985)
0.19 [−0.15, 0.53]
Ch Kiran kumar 2021 (SU009)
−0.20 [−0.51, 0.12]
Random−Effects Model
0.11 [−0.05, 0.27]
−1
−0.5
0
0.5
1
Signed standardized error
