# Supplementary material for: Accuracy of Photoplethysmography-Derived Pulse Rate Variability Compared with Electrocardiography-Derived Heart Rate Variability: A Systematic Review and Meta-Analysis
Source: Sensors (Basel). 2026 Aug 17;26(16):5192. doi: 10.3390/s26165192 (PMC13517957; doi:10.3390/s26165192)
Supplement: Supplementary file 1 [file sensors-26-05192-s001.zip › Supplementary_Figure_S2.pdf]

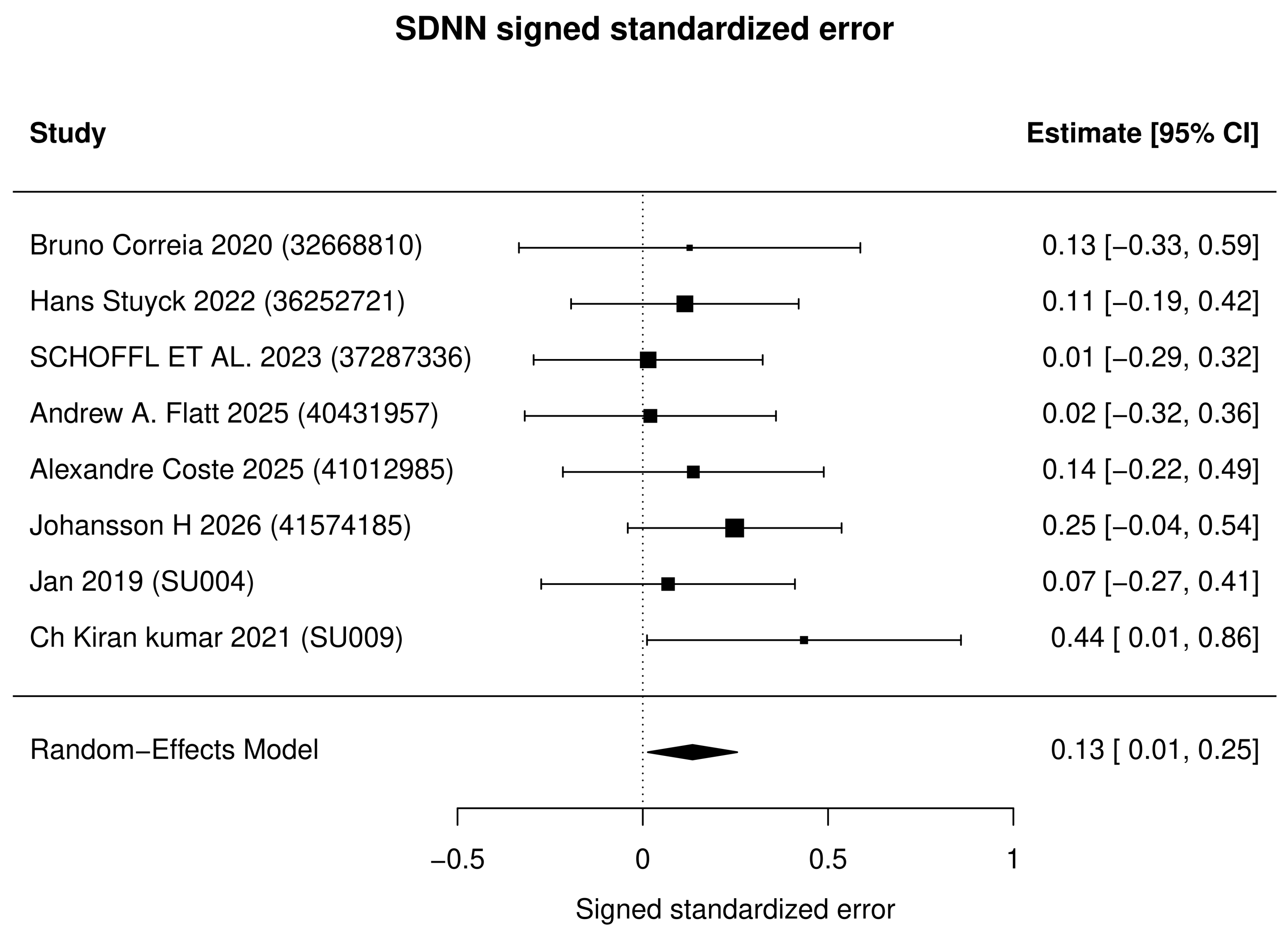
SDNN signed standardized error
Study
Estimate [95% CI]
Bruno Correia 2020 (32668810)
0.13 [−0.33, 0.59]
Hans Stuyck 2022 (36252721)
0.11 [−0.19, 0.42]
SCHOFFL ET AL. 2023 (37287336)
0.01 [−0.29, 0.32]
Andrew A. Flatt 2025 (40431957)
0.02 [−0.32, 0.36]
Alexandre Coste 2025 (41012985)
0.14 [−0.22, 0.49]
Johansson H 2026 (41574185)
0.25 [−0.04, 0.54]
Jan 2019 (SU004)
0.07 [−0.27, 0.41]
Ch Kiran kumar 2021 (SU009)
0.44 [0.01, 0.86]
Random−Effects Model
0.13 [0.01, 0.25]
−0.5
0
0.5
1
Signed standardized error
