# Supplementary material for: Accuracy of Photoplethysmography-Derived Pulse Rate Variability Compared with Electrocardiography-Derived Heart Rate Variability: A Systematic Review and Meta-Analysis
Source: Sensors (Basel). 2026 Aug 17;26(16):5192. doi: 10.3390/s26165192 (PMC13517957; doi:10.3390/s26165192)
Supplement: Supplementary file 1 [file sensors-26-05192-s001.zip › Supplementary_Figure_S3.pdf]

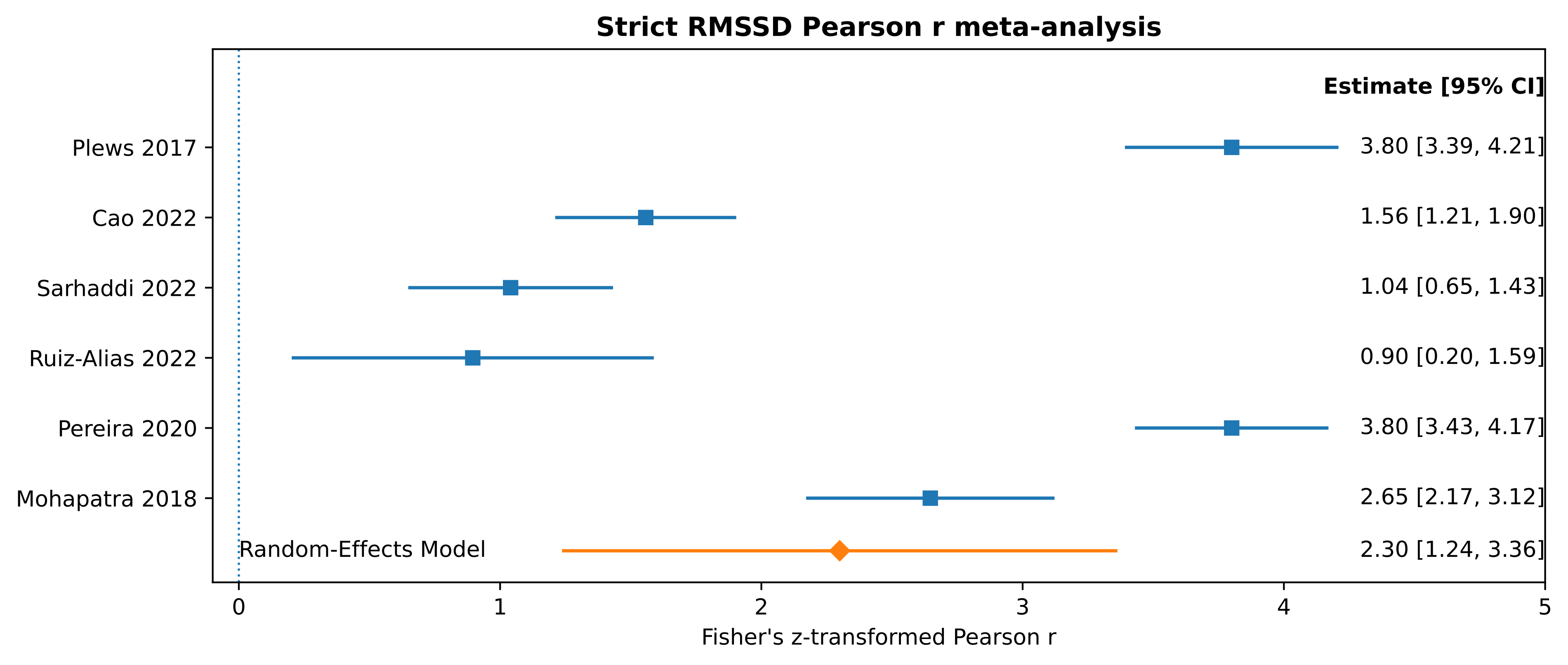
Strict RMSSD Pearson r meta-analysis
Estimate [95% CI]
Plews 2017
3.80 [3.39, 4.21]
Cao 2022
1.56 [1.21, 1.90]
Sarhaddi 2022
1.04 [0.65, 1.43]
Ruiz-Alias 2022
0.90 [0.20, 1.59]
Pereira 2020
3.80 [3.43, 4.17]
Mohapatra 2018
2.65 [2.17, 3.12]
Random-Effects Model
2.30 [1.24, 3.36]
0
1
2
3
4
5
Fisher's z-transformed Pearson r
